# PriSUD-Nordic—Diagnosing and Treating Substance Use Disorders in the Prison Population: Protocol for a Mixed Methods Study

**DOI:** 10.2196/35182

**Published:** 2022-03-23

**Authors:** Anne Bukten, Nicoline Toresen Lokdam, Ingeborg Skjærvø, Thomas Ugelvik, Svetlana Skurtveit, Roman Gabrhelík, Torbjørn Skardhamar, Ingunn Olea Lund, Ingrid Amalia Havnes, Eline Borger Rognli, Zheng Chang, Seena Fazel, Christine Friestad, Morten Hesse, Johan Lothe, Gerhard Ploeg, Anja J E Dirkzwager, Thomas Clausen, Christian Tjagvad, Marianne Riksheim Stavseth

**Affiliations:** 1 Norwegian Centre for Addiction Research University of Oslo Oslo Norway; 2 Division of Mental Health and Addiction Oslo University Hospital Oslo Norway; 3 Department of Criminology and Sociology of Law University of Oslo Oslo Norway; 4 Department of Mental Disorders Division of Mental and Physical Health The Norwegian Institute of Public Health Oslo Norway; 5 Department of Addictology First Faculty of Medicine Charles University Prague Czech Republic; 6 Department of Sociology and Human Geography University of Oslo Oslo Norway; 7 Department of Psychology University of Oslo Oslo Norway; 8 Adult Psychiatry Unit University of Oslo Oslo Norway; 9 Department of Medical Epidemiology and Biostatistics Karolinska Institutet Stockholm Sweden; 10 Department of Psychiatry University of Oxford Oxford United Kingdom; 11 University College of Norwegian Correctional Service Lillestrøm Norway; 12 Center for Alcohol and Drug Research Aarhus University Aarhus Denmark; 13 WayBack, Foundation for Life After Imprisonment Oslo Norway; 14 Directorate of Norwegian Correctional Service Lillestrøm Norway; 15 Netherlands Institute for the Study of Crime and Law Enforcement Amsterdam Netherlands

**Keywords:** substance use disorders, prison, criminal justice, epidemiology, mixed methods, harm reduction, treatment

## Abstract

**Background:**

A large proportion of the prison population experiences substance use disorders (SUDs), which are associated with poor physical and mental health, social marginalization, and economic disadvantage. Despite the global situation characterized by the incarceration of large numbers of people with SUD and the health problems associated with SUD, people in prison are underrepresented in public health research.

**Objective:**

The overall objective of the PriSUD (Diagnosing and Treating Substance Use Disorders in Prison)-Nordic project is to develop new knowledge that will contribute to better mental and physical health, improved quality of life, and better life expectancies among people with SUD in prison.

**Methods:**

PriSUD-Nordic is based on a multidisciplinary mixed method approach, including the methodological perspectives of both quantitative and qualitative methods. The qualitative part includes ethnographic fieldwork and semistructured interviews. The quantitative part is a registry-based cohort study including national registry data from Norway, Denmark, and Sweden. The national prison cohorts will comprise approximately 500,000 individuals and include all people imprisoned in Norway, Sweden, and Demark during the period from 2000 to 2019. The project will investigate the prison population during three different time periods: before imprisonment, during imprisonment, and after release.

**Results:**

PriSUD-Nordic was funded by The Research Council of Norway in December 2019, and funding started in 2020. Data collection is ongoing and will be completed in the first quarter of 2022. Data will be analyzed in spring 2022 and the results will be disseminated in 2022-2023. The PriSUD-Nordic project has formal ethical approval related to all work packages.

**Conclusions:**

PriSUD-Nordic will be the first research project to investigate the epidemiology and the lived experiences of people with SUD in the Nordic prison population. Successful research in this field will have the potential to identify significant areas of benefit and will have important implications for ongoing policy related to interventions for SUD in the prison population.

**International Registered Report Identifier (IRRID):**

DERR1-10.2196/35182

## Introduction

Globally, more than 30 million people are released from prisons each year [1], and the number is increasing [2,3]. Among people in prison, a large proportion have a history of drug use and substance use disorders (SUDs) [4,5]. A systematic review and meta-analysis found that the pooled prevalence estimate for SUD was 51% among women and 30% among men [4].

The harm caused by SUD, including the use of illicit substances as well as alcohol and other legal substances, is a significant contributor to the burden of disease [6]. Individuals with SUDs have a higher risk of premature death, ranging from 4-fold increased mortality among persons with alcohol use disorder [7] to 15-fold increased mortality among persons with opioid use disorder [8]. During 2015, 28 million years of healthy life (ie, disability-adjusted life years) were lost worldwide due to premature death and disability caused by drug use [7], with a heavier burden among socially disadvantaged groups, such as the prison population. People with SUD in prison thus constitute a group of people who are marginalized in terms of both their substance use and their incarceration, and they suffer disproportionately from poor physical and mental health, infectious diseases, social marginalization, and economic disadvantage [9].

A continuing challenge in public health is to provide services to the people who need them the most and who are the hardest to reach. Prisons may provide a unique opportunity for health interventions: a high proportion of people in prison have untreated SUD and, in prison, they are reachable for a predictable amount of time. The detection of mental health problems and SUDs, followed by adequate treatment and the introduction of harm reduction measures, may, from a public health perspective, represent a turning point in promoting SUD treatment in a highly disadvantaged group. However, the provision of health services in prison, worldwide, is characterized by large variations within a spectrum ranging from no health services to universal health coverage, with the Nordic countries being examples of the latter [10,11]. Where high-quality health services are offered to prisoners, prison is one of the few settings where health service agencies can engage in regular contact with marginalized populations that typically have precarious lifestyles when not imprisoned.

Advancing our knowledge of traditionally marginalized and understudied groups, such as people with SUD in prison, is essential to understanding social disparities in health. In addition, this is a precondition for planning the most appropriate interventions among people with SUD in prison. According to the World Health Organization (WHO), the public health importance of imprisonment is insufficiently recognized [12]. Despite the global situation characterized by incarceration of large numbers of people with SUD and the health problems associated with SUD, people in prison are underrepresented in public health research.

To address this challenge, the PriSUD (Diagnosing and Treating Substance Use Disorders in Prison)-Nordic project aims to investigate the epidemiology of SUD and explore the lived experiences of people with SUD in the Nordic prison population during three different periods: the time *before* imprisonment, the time *during* imprisonment, and the time *after* release.

To reach the aims of the PriSUD-Nordic study, we have put together a multidisciplinary research group that will analyze a wide range of existing, high-quality Nordic registry data combined with analysis of qualitative data based on ethnographic fieldwork and semistructured interviews. All the scientific aims have interlinking health and methodological perspectives as well as work packages (WPs). The WPs are related to (1) epidemiology (WP I), (2) risk assessment (WP II), and (3) qualitative methods (WP III; Table 1).

**Table 1:** Scientific aims, interlinked health and methodological perspectives, and work packages (WPs) for the periods prior to, during, and after imprisonment.

Time period and aim		Health perspective	Methodological perspective	WP^a^	
**Prior to imprisonment**					
	Investigate the prevalence of SUD^b^ among the prison population before incarceration in Scandinavia	Determine the burden of SUD-related problems and selection of persons with SUD who are in prison	Does registry data capture the prevalence of SUD across the Scandinavian countries?	I	
	Investigate the prevalence of mental and physical health problems before incarceration among the prison population	Determine the burden of mental and physical health problems and selection of persons with such problems who are in prison	Does registry data capture the prevalence of mental and physical health problems across the Scandinavian countries?	I	
**During imprisonment**					
	Test a risk assessment tool based on OxRec^c^ for identification of persons with SUD in need of treatment interventions	Identify persons in need of treatment based on routinely collected data	Improving risk assessment by testing and externally validating the OxRec instrument in the Nordic countries	II	
	Investigate and compare the availability of treatment interventions during incarceration for SUD and non-SUD populations	Determine if adequate treatment is offered in line with health policies and whether the availability of treatment for persons with SUD (first aim) differs from persons without SUD	Is registry data a valid source when estimating treatment availability in prison?	I	
	Investigate the nature, norms, mechanisms, and process of SUD treatment in Scandinavian prisons	Determine how SUD treatment is implemented and integrated into the prison context	How can qualitative analysis complement and support register data on health in prison research?	III	
**Postimprisonment**					
	Investigate and compare postrelease outcomes	Determine effects of in-prison treatment on postrelease outcomes regarding health, social welfare, and recidivism	How can variables from different countries be aligned to fully compare outcomes?	I	
	Investigate postrelease narratives among former prisoners	Describe challenges and opportunities for persons released from prison as perceived by participants in WayBack (a nonprofit foundation that helps prisoners return to society)	How can qualitative prison research guide, improve, and support implementation of health interventions in the postrelease context?	III	
	Test a risk assessment tool based on OxRec for identification of persons with a need for additional follow-up postrelease	Identify persons with a high risk of substance use postrelease to prevent relapse to substance use, crime, overdose, and other high-risk events	Improving risk assessment by testing and externally validating the OxRec instrument in the Nordic countries	II	

^a^WP I is related to epidemiology, WP II is related to risk assessment, and WP III is related to qualitative methods.

^b^SUD: substance use disorder.

^c^OxRec: Oxford Risk of Recidivism Tool.

## Methods

### Overview

Prisons are situated in complex social, cultural, and political contexts and are dependent on social, structural, and historical factors. The experience of imprisonment is shaped by the characteristics of the individual prisoners as well as relational, structural, and regime factors. This makes a multidisciplinary mixed methods approach relevant, as it includes the methodological perspectives of epidemiological quantitative methods and ethnographic qualitative methods. Mixed methods enable investigators to integrate qualitative research and qualitative data conceptually and analytically with traditional epidemiological and quantitative research methods to facilitate translation. Mixed methods will help us understand not just whether an intervention works but how, why, for whom, and under what circumstances it works [13].

### Epidemiological Approach (WP I)

A large part of the project will be based on data from nationwide public registries. This approach has several advantages: low costs, it covers the entire population, and it provides longitudinal data with controllable attrition [14-16]. The methodology—linking data through personal identification numbers to construct rich longitudinal data sets—is an important feature of PriSUD-Nordic. Typically, individual data are collected either in clinical studies, with limited follow-up time to measure posttreatment outcomes, or from registries only, excluding patient-reported outcomes. Treating these data sources as complimentary will harness the strengths of both. When performing multinational studies, problems concerning different data custodians are common: ethical and cross-jurisdictional data-sharing restrictions prevent the direct sharing of individual-level data. To account for these issues, PriSUD-Nordic will use a two-step, individual participant data meta-analysis (IPDMA) [17], described in detail in the Data Analysis Plan section.

### Risk Assessment (WP II)

Big data and machine learning are hot topics in health and medical research [18]. With increasing amounts of data available, new opportunities for statistical analysis arise. One promising method is using risk assessment tools, often referred to in medicine as prognostic models, prediction models, prediction rules, or risk scores [19]. Using data that are routinely collected among the population in question allows risk assessments tools that are used as adjuncts to be employed as practical and easy-to-use guides; these can assist clinicians, other health personnel, or prison staff in decision-making, raising the ceiling of expertise, and potentially enabling more evidence-based approaches to delineate treatment pathways.

We intend to complement the epidemiological research with a translational approach in which we test an evidence-based and scalable risk assessment tool based on the Oxford Risk of Recidivism Tool (OxRec), which was developed by the Forensic Psychiatry and Psychology group at the University of Oxford [20]. This tool requires new external validations in Nordic samples. We will test the performance of this prediction tool in new cohorts of released prisoners and consider whether it needs recalibration. In addition, we will examine how such a tool, if the external validation is promising, can be translated into practice and, in particular, whether it can be used to identify prisoners at risk of reoffending who need additional substance use treatment or other interventions, such as more regular follow-up or links with community health services.

### Qualitative Approach (WP III)

Qualitative health research methods are underused in public health research [21]. It is well established in penological research that prisons provide an environment often characterized by a series of lacks, including the lack of predictability, autonomy, and purpose [22,23]. When investigating the epidemiology of SUD among people in prison using longitudinal registry data, it is, therefore, highly relevant to take the specific context of the prison into account. Two methods will be at the center of the qualitative research: ethnographic fieldwork and semistructured interviews. The focus will be on identifying challenges to successful treatment in prison and on the prison as a specific arena for treatment interventions.

Qualitative methods can provide background and depth to epidemiological and statistical analysis, which may be helpful when developing hypotheses and research design. Qualitative analysis can also help validate or challenge the interpretation of quantitative results by developing knowledge of processes, mechanisms, and explanatory models behind the results. In addition, qualitative methods can inform the development and implementation of interventions, guidelines, and recommendations that may result from the research project. Finally, qualitative approaches will provide increased insight into the combination of, or conflict between, health and welfare-oriented goals (ie, treatment, increased health, and well-being) on the one hand, and penal goals in prison settings (ie, punishment, control, security, and retribution) on the other hand.

### Research Opportunity in Nordic Prison Settings

The Nordic countries are in an ideal position to perform world-class quality research on substance-related public health challenges. This is partly because all the Nordic countries collect individual-level data in the form of various national registries, including rich health and social services data. The countries have publicly financed health care, available to those who need it regardless of their financial situation. The similarities in societal development across the Nordic countries makes the Nordic region ideal for comparative studies within health and SUD. By investigating postrelease outcomes regarding health, social welfare, and recidivism, the output from PriSUD-Nordic will help us understand what characterizes best-practice interventions.

The Nordic countries have enormous potential for synergy, with strong health care registers, publicly owned universities and university hospitals, and a high appreciation for medical research among the public and politicians. In the recent Nordic White Paper on Medical Research, national registries were identified as a specific area where coordinated actions and determined cooperation could bring the Nordic region into a unique, global leadership position [24]. Because of all the benefits, Norwegian and Nordic research councils are currently promoting the use of national registries [24]. PriSUD-Nordic will use the full potential of these registries by applying a methodology designed specifically for multinational studies.

### Study Population and Data Sources

The Scandinavian prison cohorts will include approximately 500,000 individuals. The Norwegian prison cohort (approximately 100,000 individuals) includes all people in Norwegian prisons during the period from 2000 to 2019. The Danish prison cohort (approximately 250,000 individuals) includes people released from prison or on probation during the period from 2000 to 2012. The Swedish cohort (approximately 150,000 individuals) includes all people released from prison or on probation during the period from 2000 to 2013. All the national cohorts are drawn from the national prison registries and will be linked to (1) national cause of death registries, (2) national prescription databases (excluding the Norwegian cohort), (3) national patient registries, (4) police and crime registries, and (5) data on socioeconomic conditions. See Table 2 for a more detailed description of the national registries.

**Table 2:** Types of registries and data and their descriptions.

Type of registry or data	Description^a^
National prison registries	National prison registries include a range of personal data on people imprisoned in each country, including age, gender, convictions and sentences, and the actual time spent in prison. The registries also include some information on participation in correctional interventions.
National cause of death registries	National cause of death registries include the cause of death based on the ICD-10^b^. The registries include all residents of the country at the time of death. The registries are based on death certificates and information that is coded at a national level.
National prescription databases	National prescription databases contain information on all prescription drugs, whether reimbursed or not, dispensed by pharmacies to individual patients. All drugs are classified according to the Anatomical Therapeutic Chemical classification system.
National patient registries	National patient registries include information on all patients receiving hospital-level care in both inpatient and outpatient facilities and acute and emergency services for mental and somatic illnesses. The national patient registries also include birth date, county of residence, date of admission, date of discharge, and primary and secondary diagnoses, according to the ICD-10.
Police and crime registries	Police and crime registries include information on all registered criminal cases, including identified offenders. They provide data on several prosecuting decisions, such as formal charges leading to conviction, formal charges leading to acquittal, and fines. In addition, these registries contain dates for offences and convictions.
Data on socioeconomic conditions	Data on socioeconomic conditions include information on employment, income, and social benefits.

^a^The registry descriptions are intended as an overview and may vary across countries.

^b^ICD-10: International Classification of Diseases, Tenth Revision.

### Data Analysis Plan

Evidence from randomized controlled trials is rarely available for studies on the treatment of SUDs, as the randomized controlled trial design has ethical challenges related to harmful substances. Therefore, most studies on SUDs are observational. In such studies, associations between exposure and outcome can be explained by true causation, reverse causation, or confounding. Methods to support causal inference in observational studies are required. The PriSUD-Nordic project will adopt newly developed causal inference methods, such as nearly saturated propensity score matching, instrumental variable methods, and inverse probability weighting [25].

The quantitative part of the study is a linked registry-based cohort study. All data will be analyzed using the two-step IPDMA method. Methodologically, it allows for consistent inclusion and exclusion criteria across countries and has been successfully applied to other studies [17,26]. This method has several advantages. Because data are initially analyzed locally, we remove hurdles associated with sharing sensitive individual-level data across local jurisdictions. Thus, local investigators are provided with an opportunity to convey the nuances of data. It allows for consistent adjustment for confounding factors that may explain differences in findings across countries; in addition, it increases the clinical relevance of findings by providing the opportunity to explore clinical questions that cannot be answered by the individual countries alone [17].

The interviews will be conducted following a semistructured interview guide, based on a scoping review of the current knowledge within the field. Some of the overall themes in the interview guide may include social relations, support and marginalization, health and addiction, and motivation for treatment. A combination of thematic and inductively developed analytic codes will be employed. The focus will be on identifying challenges to successful treatment and the prison as an arena for treatment interventions.

### Ethics Approval

People in prison form a disadvantaged group in a coerced setting that carries a heavy burden of problems. This requires increased awareness of ethical boundaries by investigators and research personnel and highlights the need for scientific knowledge about the group and their situation. No prisoners will be denied treatment or experience a reduction in the quality of treatment as a result of the research project. All efforts to ensure that data are treated confidentially and in accordance with existing legislation for research data will be followed, and the project will be conducted according to the Declaration of Helsinki.

WP I and WP II will be based on registry data from Norway, Sweden, and Denmark. The Norwegian registry linkage has been approved by the Regional Committees for Medical and Health Research Ethics (REC ID 2012/1401, 29513), the Norwegian Centre for Research Data (NSD ID 847562), and the Data Protection Officer at the Faculty of Medicine at the University of Oslo. The Swedish data linkage has been approved by the Regional Ethical Review Board in Stockholm (Dnr 2013/862–31/5) and has already been obtained. In Denmark, registry data can be used for research without ethical approval. To overcome the obstacles associated with sharing individual-level data across jurisdictions, data will be analyzed separately in each country. All data will be stored according to local regulations. All dissemination resulting from the study will contain group-based information. Thus, no individual participants will be identifiable.

The national registry studies (WP I and WP II) have been approved for exemption from the consent requirement. WP III is based on ethnographic fieldwork and semistructured interviews. This part of the project has been approved by the Norwegian Centre for Research Data (NSD ID 964221). Informed written consent must be obtained from all participants.

### User Involvement

User involvement will be integrated into the steering committee and the research group, which is represented by clinicians, policy makers, and user organizations. The Norwegian user organization WayBack [27] provides services in prison and helps build prosocial networks postrelease to prevent relapse into crime and drug use. WayBack will play an essential role in planning the project, discussing research questions for the papers in the project, and recruiting participants to interview (WP III). WayBack will also be involved in our plans to disseminate the study results to participants and the relevant wider communities (eg, choosing what information and results to share, when to share them, and in what format).

## Results

PriSUD-Nordic was funded by The Research Council of Norway in December 2019, and funding began in 2020. Data collection is ongoing and will be completed in the first quarter of 2022. Data will be analyzed in spring 2022 and the results will be disseminated in 2022-2023.

## Discussion

### The Potential Impact of the Proposed Research

If our project proves to be successful, the knowledge and outputs generated from this project can provide new insight in order to solve challenges related to the United Nations’ Sustainable Development Goals. For instance, Goal 3 (Good Health And Well-being) includes Target 3.5 (Prevent and Treat Substance Abuse), which is to “strengthen the prevention and treatment of substance abuse, including narcotic drug abuse and harmful use of alcohol.”

An essential part of PriSUD-Nordic is developing organizational and methodological novelty to run a multi-organizational, multidisciplinary, and multinational research study. PriSUD-Nordic aims to develop new knowledge that contributes to better mental and physical health, improved quality of life, and better life expectancies among people with SUD in prison. More specifically, we anticipate that the aims outlined in the proposal will impact (1) establishment of a new knowledge base, (2) identification of treatment gaps and potential discrimination in treatment, and (3) provision of best-practice interventions.

### Establishing a New Knowledge Base

To prevent or promote anything effectively, we first need to *identify* the risk factors and characteristics associated with a higher likelihood of specific outcomes. In the case of harmful substance use as an outcome, prevention must consider that risk factors related to SUD vary according to *different social and political contexts* and *over the individual life course.* Preventing further development of SUD requires interventions to meet the target group’s needs and consider vulnerable transitions in life, such as cycling in and out of prison.

Although several studies have described the prison population before entering prison [28,29], the complexity of SUD, along with other health-related problems and lifestyle factors, has not yet been addressed. There is an increasing recognition that lifestyle factors can be modified to improve health outcomes directly [30]. However, while many countries have established and implemented policies and interventions that address smoking, alcohol, nutrition, and physical activity among nonclinical groups, these lifestyle factors have been addressed less frequently among the prison population. Within these groups, unmet needs must be identified, and user-acceptable and user-accessible interventions must be further developed and implemented. The output from PriSUD-Nordic will be a new and improved knowledge base for fitting best-practice interventions for the prison population.

### Identifying Treatment Gaps and Potential Discrimination in Treatment

Lives lost that are attributable to morbidity and mortality resulting from all causes of substance use have increased in the past decade [31], and data from the WHO suggest that only 7% of those with past-year SUDs received even minimally adequate treatment [32]. Among the prison population, the proportions are even smaller. This illustrates that mental health and the addiction field have lagged behind other areas of medicine in terms of resources for treatment and research, and the public health goal of reducing the world’s drug problems cannot be achieved without addressing SUDs with the same scientific commitment with which physical problems are addressed [33].

However, underuse of treatment can also result from the extensive and deeply seated stigmatization of substance users. Despite having poor health, many do not seek or receive health services [34]. Others receive lower-quality services and are judged as least deserving of health care [35]. This aspect is highlighted in the United Nations General Assembly Special Session (UNGASS) resolution, which aims to eliminate stigma and discrimination toward individuals with SUDs [36]. By investigating the need and availability of treatment interventions during incarceration based on the preprison burden of disease, the output from PriSUD-Nordic could identify treatment gaps and potential discrimination in treatment offered.

### Providing Best-Practice Interventions

According to the recent UNGASS resolution, a comprehensive public health approach should offer accessible, evidence-based prevention, treatment, and recovery options during and following incarceration [37]. According to a seminal Lancet report, receiving treatment in prison is a *human right* [38].

The Nordic countries are in an ideal position to perform world-class quality research on today’s most pressing substance-related public health challenges. This is, in part, because all the Nordic countries collect individual-level data in the form of various national registries, including health and social services data. The Nordic similarities in terms of societal development makes the Nordic region ideal for comparative studies within health and SUD. Research across the Nordic countries and within Europe has a great advantage, as findings may be compared between nations. By investigating postrelease outcomes regarding health, social welfare, and recidivism, the output from PriSUD-Nordic may contribute to the provision of best-practice interventions.

## Appendix

Multimedia Appendix 1 Peer-review report.

## Abbreviations

IPDMA: individual participant data meta-analysis
OxRec: Oxford Risk of Recidivism Tool
PriSUD: Diagnosing and Treating Substance Use Disorders in Prison
SUD: substance use disorder
UNGASS: United Nations General Assembly Special Session
WHO: World Health Organization
WP: work package

## Availability of Data and Materials

This population study will be based on individual-level data from the Norwegian Prison Registry, which is held by the Directorate of Norwegian Correctional Service, and the Norwegian Cause of Death Registry, which is held by the Norwegian Institute of Public Health. The ethical approval of this research project does not include permission to publicly share the raw data. Qualifying researchers can apply for access to relevant data through the Norwegian Institute of Public Health and the Directorate of Norwegian Correctional Service upon approval from the Regional Committees for Medical and Health Research Ethics.
