# Supplementary material for: PriSUD-Nordic—Diagnosing and Treating Substance Use Disorders in the Prison Population: Protocol for a Mixed Methods Study
Source: JMIR Res Protoc. 2022 Mar 23;11(3):e35182. doi: 10.2196/35182 (PMC8987966; doi:10.2196/35182)
Supplement: Multimedia Appendix 1 [file resprot_v11i3e35182_app1.pdf]

Forskningsrådet

Assessment of grant application submitted to the Research Council of Norway

Grant application

Project number	301535
Project title	PriSUD-Nordic: Intervening substance use disorders in the prison population - A public health opportunity
Project manager	Bukten, Anne
Project owner	Institutt for klinisk medisin
Application type	Researcher Project /
Programme/Activity	Bedre helse og livskvalitet
Case officer	Torbjørg Øyslebø

Confirmation

By completing and submitting this form, I / we confirm the following (applies for the individual referee or the referee panel):

- I/We have no conflicts of interest that would prevent me/us from conducting this assessment. See Regulations on Impartiality and Confidence in the Research Council of Norway.	Yes
- I/We have read and understood both the criteria I/we have been asked to use for assessing the application and the description of the scale of marks. The scale of marks is to be applied as an absolute scale, i.e. marks are to be determined for each grant application independently and not relative to other applications that the panel/referee is assessing.	Yes
- I/We understand and accept the guidelines for assessing applications for the Research Council of Norway. See Guidelines for referees/panels who assess applications for the Research Council of Norway.	Yes
- I am/We are qualified to conduct this assessment.	Yes

Summary of marks

Criterion	Mark
Excellence	5
Impact	6
Implementation	5
Overall assessment of the referee/panel	5

Criteria

Excellence

.

The extent to which the proposed work is ambitious, novel, and goes beyond the state of the art
* Scientific creativity and originality.
* Novelty and boldness of hypotheses or research questions.
* Potential for development of new knowledge beyond the current state of the art, including significant theoretical, methodological, experimental or empirical advancement.

The quality of the proposed R&D activities
* Quality of the research questions, hypotheses and project objectives, and the extent to which they are clearly and adequately specified.
* Credibility and appropriateness of the theoretical approach, research design and use of scientific methods. Appropriate consideration of interdisciplinary approaches.
* Where relevant, the extent to which appropriate consideration has been given to ethical issues, safety issues, gender dimension in research content, and appropriate use of stakeholder/user knowledge.

The proposed project will investigate persons with substance use disorder in prison prior and during prison as well as post-release. The project addresses an important scientific and public health issue and includes 3 work-packages: epidemiology, risk assessment and a qualitative component. The combination of Registry data and qualitative methods is particularly strong. The Registry data, in particular, and the construction of synthetic cohorts is a considerable strength and offers opportunities for unique insights. Similarly, WP2, based on registry data, proposes the use of relatively sophisticated risk prediction models and has considerable potential. The project is thus ambitious and it holds potential for developing new knowledge, especially because of the possibility of synthesizing pre-prison, during prison and post-release data.

The project has a small number of shortcomings. The screening and risk assessment tool OxRec is not defined. Why it is this particular tool that the project wants to apply? More importantly, it is unclear if all WPs will be conducted in both Norway, Denmark and Sweden. For example, will WPII Aim III, and Aim QI and QII be conducted in all three countries? This is especially important since funding is only applied for to the Norwegian partner. The use of prison cohorts (box 1) needs some more reflections in relation to when prison drug treatment interventions are implemented in N, S and DK. While it is well argued why ethnographic and qualitative methods should be part of the proposed project, the arguments are not well-founded in already existing research. It is also not sufficiently described how many interviews, with whom, and where to conduct ethnographic fieldwork and for how long. More discussion of limitations of registry data (esp for 'prior to prison') in terms of identification of cases given low rate of treatment seeking, may have been useful.

Selected mark : 5 - Very good
The proposal addresses the criterion very well. A small number of shortcomings are present.

Impact

.

Potential impact of the proposed research
* The extent to which the planned outputs of the project address important present and/or future scientific challenges.
* If relevant with respect to the project objectives, the extent to which the planned outputs will address UN Sustainable development goals or other important present and/or future societal challenges.
* The extent to which the potential impacts are clearly formulated and plausible.

Communication and exploitation
* Quality and scope of communication and engagement activities with different target audiences, including relevant stakeholders/users.

The project holds potential to have societal and scientific impact, and these are well described. It is well argued how the project taps into UN's sustainability goals and how the creation of a new knowledge base will be able to inform future prison based and post release interventions.

The project will use different outlets and channels to disseminate results, and these are well described. Including scientific journals, newsletters to reach non-academic audiences, webpages, social media, etc. They project will also produce popular scientific reports to distribute in all Scandinavian prisons.

Selected mark : 6 - Excellent
The proposal successfully addresses all relevant aspects of the criterion. Only minor shortcomings are present.

Implementation

.

The quality of the project manager and project group
* The extent to which the project manager has relevant expertise and experience, and demonstrated ability to perform high-quality research (as appropriate to the career stage).
* The degree of complementarity of the participants and the extent to which the project group as a whole assembles the necessary expertise needed to undertake the research effectively, and provides added value.

The quality of the project organization and management
* Effectiveness of the work plan, including the extent to which resources assigned to work packages are aligned with project objectives and deliverables.
* Appropriateness of the allocation of tasks, ensuring that all participants have a valid role and adequate resources in the project to fulfil that role.
* Appropriateness of the proposed management structures and governance.

The project manager is according to her age and leaves an experienced researcher with a good track record of both publications and research project. The research team is interdisciplinary and holds the expertise needed to implement the project's aims and WPs. There is an international steering committee connected to the project consisting of esteemed researchers within the field and representatives from user organizations and health administrations.

There is a well developed work plan and all elements of the projects seems to be included, despite the lack of details concerning WPIII.

Overall, the comparative aspect of the project is a real strength to the proposed project.

Selected mark : 5 - Very good
The proposal addresses the criterion very well. A small number of shortcomings are present.

Overall assessment of the referee/panel

.

Overall assessment of the referee/panel based, on the criteria Excellence, Impact and Implementation.

The project is ambitious and holds a lot of potentials because of it's cross national and mix-method approach. It is an excellent application of SUD identification and treatment in prison environment, with a strong research team. There are, however, a small number of shortcomings, especially in relation to the design and work plan, including uncertainty about WPIII and the work loads in DK and S.
The combination of registry data and qualitative methods is potentially powerfull and is a relatively sophisticated approach to risk prediction. There could also have been more discussions of limitations of registry data (esp for 'prior to prison'), including the uncertainty/unknown about range validity of data available in registries and that some of the prison cohorts are not updated, and therefore we do not have data from the past 5-7 years.

Selected mark : 5 - Very good
The proposal is very good. The criteria are very well addressed. A small number of shortcomings are present.

Special points to consider

Comments to special points to consider
